# Miscellany

**Published:** 1888-05

**Authors:** 


### MISCELLANEOUS.

The *Philadelphia Medical Times* says, in regard to substitution and adulteration, it must be admitted that in numerous cases the charge is a true one, and the evil is of growing dimensions. With the reduction in the margin of profits caused by the fierce business competition of the present day comes the temptation to adulterate, or substitute inferior quality. No condemnation can be too severe for the man who thus trifles with human life; and if he cannot carry on his business honestly, he had better abandon it and seek some other occupation.

Again, the outcry is made that the physician is too apt to prescribe various remedies, more or less proprietary in character, put up by large manufacturing concerns and introduced by skilled advertising, and thus require the druggist to carry an endless variety of such articles in stock, many of which are seldom or only once called for, and thus remain a dead loss to the proprietor. But is the physician much to blame? True, he is sometimes imposed upon by the bland and suave canvasser, and the glowing printed indorsements of his professional brethren in favor of some new remedy—*vide* stenocarpine. But when he sees remedies in convenient and compact shape, of appearance much more elegant than those he can procure from the corner druggist, and of at least equal efficacy, is it to be wondered that he should prefer X., Y., or Z.'s manufactures to the oftentime imperfectly prepared remedies of the pharmacopœia.

And here let a word be said for that much abused class, the modern manufacturers of pharmaceutical specialties. The medical and pharmaceutical professions owe to them a great debt. It is their industry and their capital which have developed the perfection of the coated pill and the compressed tablet, the pancreatic ferment and the scale pepsin, the smooth and palatable cod liver oil emulsion and the perfected extracts of malt. To their energy do we owe the modern methods of treating disease with pre-digested and concentrated foods—a plan which has been the means of prolonging many valuable lives. They have spread the fame of American pharmacy over the entire globe, and established its supremacy against all competitors; therefore let them receive at least just recognition and honor for their labors.

---

THE SUMMER SESSION.—Tradition in medical schools disparages the summer session. The 1st of October is the student's New Year's Day. At once he begins his work in the dissecting room, and, with the exception of a few days' holiday at Christmas, his labors are continued till the end of March. The new requirements of the conjoint examining board will give greater importance to the summer session. The medical schools are shaping their arrangements in accordance with the new order. When the student enters in May, he will enjoy the advantage of getting used to hospital life, and will be able to spend part of his summer vacation in learning osteology. At the end of September he will know how to ensure a "part" in due season. It is, therefore, satisfactory to learn that the warden of the College at St. Bartholomew's Hospital has issued a notice encouraging the entrance of freshmen in May. The notice advises a student who has passed a preliminary examination in arts early in the year (and has not commenced medical study) to enter in May, and to pursue his studies as follows:

1. In his first summer session to attend chemistry lectures and practical chemistry, with lectures on chemical physics. At the end of this session to pass the examination of the examining board in England of the Royal College of Physicians and Royal College of Surgeons in these subjects. He thus goes from his chemistry lecture to the practical study of the subject, and has the direct stimulus in his work of a public examination at the end of the session. 2. In his first winter session to attend anatomy lectures and dissect; to attend physiology lectures and practical physiology classes. At the end of his first winter session to pass the examination of the examining board in elementary anatomy and physiology. 3. In his second summer session to attend materia medica lectures and practical pharmacy. At the end of this summer session to pass the examination of the examining board in materia medica and pharmacy. 4. In his second winter session to dissect, and to study practical physiology, and to attend the advanced course of lectures on anatomy. He may with advantage also attend a second course of physiology lectures. At the end of his second winter session, to pass the second examination of the examining board in anatomy and physiology. Although systematic clinical work is necessarily deferred until after a student has passed his examinations in anatomy, physiology, and the other elementary subjects, he should from the beginning of his career, take every opportunity of learning the rudiments of practical medicine and surgery by attendance, as opportunities occur to him, in the wards, out-patient rooms, and the *post-mortem* room.

We understand that other medical schools intend to follow the example long ago set by University and King's Colleges, and more recently by St. Mary's, and to encourage the entry of new students in summer.—*The British Medical Journal.*

---

An English Sanitarium in Brazil.—Among health-resorts beyond Europe, where invalids and tourists may find sunshine and warmth, and a dry, bracing climate, along with home comforts, San Paulo, in Brazil, has already attracted attention, says the *British Medical Journal*, of March 31, 1888. From Rio to San Paulo is a railway ride of thirteen hours, but from the port of Santos it is only a three hours' run. Santos is twenty-eight days from New York. San Paulo has now only between 40,000 and 50,000 inhabitants, among whom are Brazilians, Portuguese, Germans, Italians, French, English, and Americans. The climate, both winter and summer, is delightful, and has been recommended for pulmonary invalids. The atmosphere is dry and exhilarating, and the barometric range so remarkably limited that it does not exceed .75 inch throughout the year. The average maximum temperature in the hottest month, January, is 80° Fahr. in the shade; in the coldest month, July, 72°. The average minimum night temperature in January is 64°; in July, 49°. The number of days on which there is brilliant sunshine for the whole or the greater part of the day averages 235 per annum, and such days are pretty equally distributed throughout the year. San Paulo has no unhealthy season of the year. Invalids are recommended to spend both seasons there, and it is uncertain which is most beneficial; but they that want sea-bathing and a change can go to Santos for bathing in June or July. Two miles from the city a sanitarium has been built, is well drained and supplied with excellent water. An English physician is resident in San Paulo, and is an able guide and adviser to those that go there for health.

### RE-VACCINATION.

THE AGE FOR RE-VACCINATION.—The local government board has issued an order, dated February 3, 1888, amending former regulations as to the age at which re-vaccination may be furnished at the public expense. The new order provides that re-vaccination by a public vaccinator may be performed when the applicant has attained the age of twelve years (instead of fifteen years as heretofore), or in case of imminent danger from small-pox, at ten years (instead of twelve as heretofore), and has not before been successfully vaccinated.

The order further provides, with reference to children in public institutions, that the medical officer may re-vaccinate any one under ten years of age, whose primary vaccination he deems to have been inadequate, and who has not before been successfully re-vaccinated.

THE HYGIENIC SIGNIFICANCE OF RE-VACCINATION.—Dr. R. Gerstäcker, in a paper with the above title, gives some valuable data compiled from the reports of the Imperial Board of Health, and also from the report of the vaccination committee. From their tables it appears that the mortality from small-pox in Prussia, formerly differing but little from that of other countries, has fallen to a minimum under the operations of the vaccination law, so that small-pox may now be considered as having disappeared, except in some frontier districts, while Austria, with her defective regulations as to vaccination, and still more so as to re-vaccination, suffers severely from small-pox. Dr. Gerstäcker presents the following table as to the mortality from small-pox in London and in Berlin, with the comment that, while London enforces vaccination of the children, it has not enforced re-vaccination. He attributes the difference in the relative mortality from small-pox in the two cities to these facts.

DEATHS FROM SMALL-POX PER 100,000 INHABITANTS.

| | 1875 | 1876 | 1877 | 1878 | 1879 | 1880 | 1881 | 1882 | 1883 |
|---|---|---|---|---|---|---|---|---|---|
| In London........ | 1.3 | 20.8 | 71.0 | 38.8 | 12.1 | 12.5 | 61.9 | 11.1 | 3.4 |
| In Berlin ....... | 5.2 | 1.8 | 0.4 | 0.8 | 0.7 | 0.8 | 4.7 | 0.4 | 0.3 |

The percentages in the following table, for a period of six years, also present further evidence of the advantages of re-vaccination.

BAVARIA.

| | Cases of Small-Pox. | Deaths from Small-Pox. | Unvaccinated. | | | Vaccinated. | | | Re-vaccinated. | | |
|---|---|---|---|---|---|---|---|---|---|---|---|
| | | | Cases. | Deaths. | Percentage of Deaths. | Cases. | Deaths. | Percentage of Deaths. | Cases. | Deaths. | Percentage of Deaths. |
| 1879 | 145 | 22 | 17 | 7 | 41.1 | 110 | 15 | 13.6 | 18 | 0 | 0. |
| 1880 | 404 | 58 | 27 | 10 | 37.0 | 336 | 43 | 12.8 | 41 | 5 | 12.2 |
| 1881 | 559 | 78 | 56 | 27 | 48.2 | 466 | 48 | 10.3 | 37 | 3 | 8.1 |
| 1882 | 468 | 71 | 33 | 15 | 45.5 | 349 | 51 | 14.6 | 86 | 5 | 5.8 |
| 1883 | 247 | 34 | 11 | 5 | 45.4 | 198 | 29 | 14.6 | 38 | 0 | 0. |
| 1884 | 63 | 8 | 4 | 2 | 50.0 | 51 | 5 | 9.8 | 8 | 1 | 12.5 |
| Average.. .. | | | | | 44.6 | | | 12.6 | | | 6.1 |

*Boston Medical and Surgical Journal.*

---

THE TOXIC PRINCIPLE OF EXPIRED AIR.—Drs. Brown-Séquard and d'Arsonval have presented a communication to the Academy of Sciences, intended to demonstrate the existence of a toxic principle in air expired from the lungs of men, or other animals. The facts hitherto known are the following :

(1) Expired air almost always, if not always, contains ammonia, but in a quantity insufficient to account for the injurious action of such air.

(2) Expired air contains a small quantity of organic matters, which, if they are not already decomposed on leaviug the air-passages, yet have a marked tendency to rapid change.

(3) Confined air, charged with pulmonary exhalations, is not injurious, on account of the carbonic acid contained in it. Atmospheric air to which 1 per cent. of carbonic acid has been added causes but little inconvenience, while expired air containing no more carbonic acid is extremely noxious,

Experiments were conducted in which rabbits were treated with injections of water containing the toxic principle of pulmonary mucus.

The primary effects were dilation of the pupils; slowing of the respiration; paralysis, especially of the lower extremities; rapid diminution of temperature from half a degree to five degrees C.

In larger doses there were more pronounced symptoms: trembling and general convulsions; attitude *recroquevillée;* choleriform diarrhœa, continuing till death ensued, in three or four hours after the injection.

The conclusions of the experimenters are that—

1. The lungs of men, of dogs, and of rabbits in health evolve an extremely energetic poison, which is continuously exhaled with the expired air.

2. It is probable, if not certain, that it is this poisonous agent which makes confined air injurious.

The true prevention of danger is found in efficient ventilation.—*Boston Medical and Surgical Journal.*

---

MEDICAL SCHOOLS AND STUDY IN THE UNITED STATES.—Professor EMILIO DE ROSSI, of the University of Rome, Italy, and an officer in the Ninth International Medical Congress, read a paper before the Royal Academy of Rome, on his return home from the congress, on "Medical Schools and Special Study in the United States." He speaks, not without some expressions of surprise, of the number of medical institutions in America, and makes special mention of some of the larger and older schools, printing in full the studies in the courses of two of the oldest of the universities in this country. He was particularly surprised to find such great opportunities for special studies in medicine, in the many hospitals, clinics, and polyclinics of the country, referring more particularly to the advantages afforded to students of ophthalmology and otology, he himself being a distinguished otologist. He particularly praises one of the well-known institutions of New York for the study of ophthalmology and otology, and speaks of the great liberality in making the institution what it is. In concluding his paper he calls attention to the large number of the candidates for the doctorate that are graduated by the schools of the country, placing the percentage at 45, which is too high. In some of our schools, he says, the degree is granted too freely; our courses are too few, and too little attention is paid to general and scientific education preliminary to the study of medicine. He speaks well of the activity of the medical men of America, and the great interest that they take in medical matters of all kinds. He thinks that there should be at least one year of university study before the study of medicine is begun, and that the study of special subjects should be compulsory in our schools. As it is, he says, we have accomplished superb results with our methods of medical education.

---

AN effort is being made in London to secure for foreigners residing in England the right to practice medicine in the United Kingdom.

In the House of Commons, April 9, 1888, Mr. Arnold Morley asked whether registered medical practitioners in the United Kingdom were afforded privileges of practicing in the United States of America, and whether any steps have been taken or were in contemplation by the privy council under section 17 of "The Medical Act, 1886," in the direction of similar privileges being given to legally qualified American practioners who might be desirous of practicing medicine in the United Kingdom.

"Sir W. Hart Dyke replied that no statement had been received from the Government of the United States showing the privileges afforded in America to registered medical practitioners of the United Kingdom, nor did he find that any request had been made by the United States Government for the extension of privileges to American medical men in this country,"

It was stated, in reply, that registered medical practitioners of the United Kingdom are afforded all rights and privileges of practicing medicine in the United States on equal terms with graduates of medical schools in the United States of America, and that the same courtesy is shown members of the other learned professions, holding certificates of their qualifications from the United Kingdom, that is shown to residents of the United States under the same circumstances.

---

Sanitary Plumbing.—Medical officers of health have consistently and persistently supported the movement now on foot for the reform of plumbers' work. The medical officer of Liverpool (Dr. Taylor) has recently brought the subject prominently before the health committee of the city, and he is, we learn, now taking steps to obtain such support from the architects and plumbers of the locality as will secure the formation of a district council to carry out in Liverpool the examination and registration of plumbers on a national system inaugurated by the Worshipful Plumbers Company, London. The medical officer of Birkenhead (Dr. Vacher), who has evinced an active interest in the movement from the outset, read a paper on Defective Plumbing at the Royal Institution, Liverpool, on Monday, April 16, illustrating it by some of his own sketches of cases coming under his observation.—*The British Medical Journal.*

The Research Scholarship of the British Medical Association.—Dr. Ralph Stockman has been appointed research scholar of the British Medical Association. Dr. Stockman is assistant to the professor of materia medica in the University of Edinburgh, and received a special training in the modern methods of pharmacological and physiological research in the laboratories of Professors Schiemedeberg and Hoppe-Seyler. He proposes to continue or undertake researches on the following subjects: 1. The chemical changes which menthol, camphor, and similar bodies undergo after absorption; how far these metabolic products affect the organism, and whether they still act as antiseptics. 2. The exact mode of action of the camphor group on the heart and circulation. 3. The pharmacology of a new body which acts like digitalis. 4. The problems connected with the absorption and excretion of glycerine and of fats. 5. On the connection between chemical constitution and physiological action of some alcohols.—*The British Medical Journal.*

---

The Woman's Medical College held its commencement exercises on April 3. The following is the list of graduates:

| | |
|---|---|
| Annie Emilie Barlow | Mary Jeanette Kearsley |
| Emma Earle Chenault | Lelia Latta |
| Marie M. Côté, M. E. L. | Anna M. Lemon |
| Agnes Eichelberger | Ella M. Minnick |
| Izillah Ernsberger | Eliza Roxana Morse |
| Helen Charlotte Gilman | Louise Sedgwick |
| Harriette Amanda Howe | Mary Belle Tuttle |
| Jennie E. Jones | Harriette Florella Wolfe |

---

Hospital Appointments.—*Mercy Hospital.*—W. D. Storer, W. S. Hall, H. B. Carriel.

*St. Luke's Hospital.*—L. L. Gregory, T. B. Swartz, E. J. Schwandt, J. S. Perekhan.

*Michael Reese Hospital.*—C. B. Wagner.

*Cook County Hospital.*—J. B. Herrick, E. J. Brown, H. R. Wittwer, F. J. Hodges, C. W. Burson, M. J. Kersley, T. J. Haines, ——Lewis.

LASSAR'S OINTMENT FOR SCABIES.—LASSAR claims good results from the use of the following ointment in scabies:

Naphthol, grammes, 5 to 10.
Green soap,
Precipitated chalk,
Washed sulphur,
Lanolin āā, grammes, 25.

*Journal Cutaneous and Genito-Urinary Diseases*, February, 1888.

---

THE CHICAGO GARBAGE CREMATORY continues to work well. It seems fully to meet the anticipations of the Health Department of Chicago, and promises to afford the city relief, that was much needed, from the annoyance of garbage that, prior to its erection, had never been satisfactorily disposed of here. Should it, upon further trial, continue to work as well, it is contemplated to erect additional ones for other sections of the city.

---

LAILLER'S WASH FOR PALPEBRAL ECZEMA.—LAILLER recommends the following wash for cases of palpebral eczema:

Crystallized acetic acid.......2 parts
Glycerine..................50 "
Cherry-laurel water (distilled)200 "

To be painted on the eyelid once a day. The brush should be slightly resistent, and care should be taken in applying the mixture.—*Riforma Medica*, March 22, 1888.

---

IMPURE ANTIPYRIN.—Professor DUJARDIN-BEAUMETZ calls attention to the fact that in some cases due care is not exercised in the preparation of this drug, a certain proportion of benzine having been detected in some samples that were analyzed. He thinks that this impurity may account for some of the toxic symptoms that have been reported as occurring, after the use of antipyrin, such as eruptions, gastric troubles, and grave cerebral symptoms.

ARSENIC SUBCUTANEOUSLY IN CHOREA.—Make a mixture of equal parts of Fowler's solution and distilled water. Begin by injecting one drop into the subcutaneous cellular tissue, and increase the quantity by one drop a day until eight or ten drops are being injected, when the quantity is reduced one drop a day. If necessary repeat.—*Gazette Médicale de Montreal*, January, 1888.

---

THE POLYCLINIC IN ROME.—In February the corner-stone of the new Polyclinic to be built in Rome was laid with impressive ceremonies, including an oration by Professor Bacelli. The building will have an area of more than 160,000 square yards, and when finished will be the most thoroughly equipped building of the kind in the world.

---

POLYPUS OF THE TONSIL.—LUBLINSKI reports a rare case of polypus of the tonsil, which he found on the lower segment of the left tonsil of a patient. It was about 3.2 cm. long, and from 3 to 5 cm. broad, with its base on the back of the tongue. It was removed by means of the scissors.—*Centralbl. für Chirurgie*, No. 8, 1888.

---

THE GABRIELE BELLION PRIZE of the Académie des Sciences de Paris, will be awarded annually to the one writing the best work on, or making the most useful discovery in, human sanitation or for the advancement of the human race.

---

DR. LEOPOLD VON HOLST, for many years one of the editors of the *St. Petersburger Medicinische Wochenschrift*, and one of the leaders of the medical profession in Russia, died recently of pyæmia.

---

SMALL-POX IN HAVANA.—During the month of January, 1888, 274 persons died of small-pox in Havana, which was a decrease of 98 as compared with December, 1887.

PROFESSOR KÜSTNER has been called to the University of Dorpat, to take the place of Dr. Wyder, who has taken Runge's former chair in Halle.

---

DR. HEBRA says: The only active treatment of dermatitis papillaris consists in destruction of the diseased tissue, which is best done with Paquelin thermo-cautery.

---

THE EMPEROR WILLIAM, of Germany, died of renal colic, according to the *British Medical Journal.* He had been subject to attacks of this malady for several years.

---

REVUE MÉDICALE PHARMACEUTIQUE is a new journal edited by Dr. Apery, of Constantinople, and published in the French, Turkish, and Greek languages.

---

DR. B. FISCHER, who accompanied Dr. Koch to Egypt and India, has been appointed Privatdocent for Bacteriology in the University of Kiel.

---

THE UNIVERSITY OF BOLOGNA, Italy, the oldest in Europe, will celebrate its 800th anniversary on June 12.

---

A SANITARY convention will be held at Manistee, Mich., under the auspices of the State Board of Health, on Tuesday and Wednesday, June 6 and 7, 1888.

---

IT has recently been proposed to substitute the Greek letter Delta (Δ) for the present dram sign (ʒ) in prescription-writing.

